# Species-Specific Responses of Carnivores to Human-Induced Landscape Changes in Central Argentina

**DOI:** 10.1371/journal.pone.0150488

**Published:** 2016-03-07

**Authors:** Nicolás Caruso, Mauro Lucherini, Daniel Fortin, Emma B. Casanave

**Affiliations:** 1 Grupo de Ecología Comportamental de Mamíferos (GECM), Cátedra de Fisiología Animal, Departamento de Biología, Bioquímica y Farmacia, Universidad Nacional del Sur, Bahía Blanca, Argentina; 2 Consejo Nacional de Investigaciones Científicas y Técnicas (CONICET), Buenos Aires, Argentina; 3 Département de biologie and Centre d'étude de la forêt, Département de Biologie, Université Laval, Québec, Canada; 4 Instituto de Ciencias Biológicas y Biomédicas del Sur (INBIOSUR), Universidad Nacional del Sur and Consejo Nacional de Investigaciones Científicas y Técnicas (CONICET), Bahía Blanca, Argentina; Federal University of Parana (UFPR) – Campus Palotina, BRAZIL

## Abstract

The role that mammalian carnivores play in ecosystems can be deeply altered by human-driven habitat disturbance. While most carnivore species are negatively affected, the impact of habitat changes is expected to depend on their ecological flexibility. We aimed to identify key factors affecting the habitat use by four sympatric carnivore species in landscapes of central Argentina. Camera trapping surveys were carried out at 49 sites from 2011 to 2013. Each site was characterized by 12 habitat attributes, including human disturbance and fragmentation. Four landscape gradients were created from Principal Component Analysis and their influence on species-specific habitat use was studied using Generalized Linear Models. We recorded 74 events of *Conepatus chinga*, 546 of *Pseudalopex gymnocercus*, 193 of *Leopardus geoffroyi* and 45 of *Puma concolor*. We found that the gradient describing sites away from urban settlements and with low levels of disturbance had the strongest influence. *L*. *geoffroyi* was the only species responding significantly to the four gradients and showing a positive response to modified habitats, which could be favored by the low level of persecution by humans. *P*. *concolor* made stronger use of most preserved sites with low proportion of cropland, even though the species also used sites with an intermediate level of fragmentation. A more flexible use of space was found for *C*. *chinga* and *P*. *gymnocercus*. Our results demonstrate that the impact of human activities spans across this guild of carnivores and that species-specific responses appear to be mediated by ecological and behavioral attributes.

## Introduction

The joint impact of lack of protection, habitat alteration, and direct killing has had an especially strong effect on mammalian carnivores, reducing their diversity and occurrence [1, 2]. These threats tend to co-occur in landscapes modified by agricultural and ranching activities. Accordingly, a recent global review found a clear conservation conflict between agricultural expansion in the 21st century and carnivore decline [3]. Carnivores are particularly vulnerable to local extinction in these types of landscapes because of their comparatively large home ranges, low population numbers, and direct persecution by humans mostly due to hunting for the skin trade and predator control [4].

Both the amount of suitable habitat in a landscape and its level of fragmentation are important predictors of the distribution and abundance of biological populations [5, 6]. In addition to the indirect effect of habitat modification, direct anthropogenic impacts, such as mortality caused by vehicle impacts on roads or human-wildlife conflicts, can also affect the distribution and abundance of a species [7–9].

Empirical studies report that responses to habitat modification and fragmentation among carnivore species are mediated by a variety of factors such as dietary breadth, behavioral adaptability, habitat requirements, and interspecific interactions within carnivore guilds and with humans [4, 10]. Where natural habitats have been reduced and fragmented, the populations of specialist carnivores tend to decrease and even become endangered [11, 12]. On the contrary, generalist carnivores seem to be tolerant or even to benefit from agricultural and suburban development [13–15].

In fragmented landscapes, habitat utilization by carnivores is often affected by the structure, configuration, and size of fragments [14, 16]. For example, larger and more homogeneous habitat fragments may be able to maintain a greater number of carnivore territories of a species strongly associated to that habitat, which could be important for its conservation [17]. Conversely, some generalist species may benefit from greater areas of disturbed habitats surrounding small fragments of pristine habitat because food availability and diversity are often higher in areas associated with open farmland, urban residences, or other human-related areas [18, 19].

The expansion of the agriculture frontier and the increase of livestock activities have caused a great amount of habitat loss in different ecosystems of Argentina, and the Espinal is not an exception. This region is an arid grassland and shrubland mosaic endemic to Argentina covering 291,941 km^2^ and has been greatly affected by human activities since the 1600s, when cattle became the prominent species on the landscape. In the last decades, agriculture has contributed greatly to habitat modification. In the southernmost part of this ecoregion (13,600 km^2^), for example, the proportion of cropland increased from 31.1% to 64.2% in the last 30 years, whereas that of shrubland decreased from 64.2% to 31.8% [20]. Found today only in small patches, the original habitats of this ecoregion were once home to a great diversity of plants, birds, and mammals, including seven species of mammalian carnivores. Despite the lack of research studies, there is great concern that the destruction and modification of natural habitats has caused a reduction in the distribution and population numbers of these carnivores.

The aim of this study was to identify the effect of environmental and anthropogenic features on the occurrence of four carnivore species in a landscape of the Espinal of central Argentina, and analyze interspecific variations in responses to anthropogenic habitat modifications. The species studied varied largely in their degree of ecological specialization and adaptability, as well as on the degree of their conflict with the ranching activities that are a major source of economic income in our study area. The Pampas fox, *Pseudalopex gymnocercus*, is a 4–6 kg generalist and opportunistic mesopredator probably able to adapt to large environmental modifications and variations in the population dynamics of its prey [21, 22]. The Molina’s hog-nosed skunk (skunk, henceforward), *Conepatus chinga*, is a 1–3 kg mephitid that specializes to forage on invertebrates [23] and appears to be common in agroecosystems of Argentina [24] and Brazil [25]. On the contrary, the Geoffroy’s cat, *Leopardus geoffroyi*, and puma, *Puma concolor*, (mean body weight: 4.5 kg and 40.5 kg, respectively) are more selective in their feeding habits and less adaptable to the environmental disturbances and the changes in prey abundances caused by humans [26, 27]. Both pumas and Pampas foxes are strongly persecuted by ranchers because they also prey on livestock. The conservation status of the four studied species has been categorized as “Least Concern” at both national and global level [28–32].

In this study we aimed to test the following predictions: 1) given that the populations of pumas in our study area appear to be heavily affected by anthropogenic pressure [33], we predicted that this species would avoid the areas fragmented and modified by human activity and would be associated with the most preserved habitats. 2) A certain avoidance of areas with intense human activity was expected also for Pampas foxes, because of the strong hunting pressure on its populations in this area [34]. 3) Due to its association to open habitat [21, 35], we also expected that Pampas foxes would use grasslands intensively. 4) Because the Pampas fox is the most ecologically adaptable species of the carnivore guild of the Espinal, we expected that it would show a less marked response to landscape composition than more selective species, such as Geoffroy’s cat. 5) Based on what is known by their respective habitat preferences, we expected that skunks would make intensive use of croplands located in proximity of comparatively more densely vegetated patches [36, 37], whereas Geoffroy’s cat would show an association with the habitat with the densest cover of natural vegetation, namely the shrubland [38].

## Materials and Methods

### Ethics Statement

Relevant authorizations to carry out the ecological research were obtained from the Organismo Provincial para el Desarrollo Sostenible (authorization number 96/2015 and 98/2015) and from ranch owners.

### Study Area

**Fig 1:**
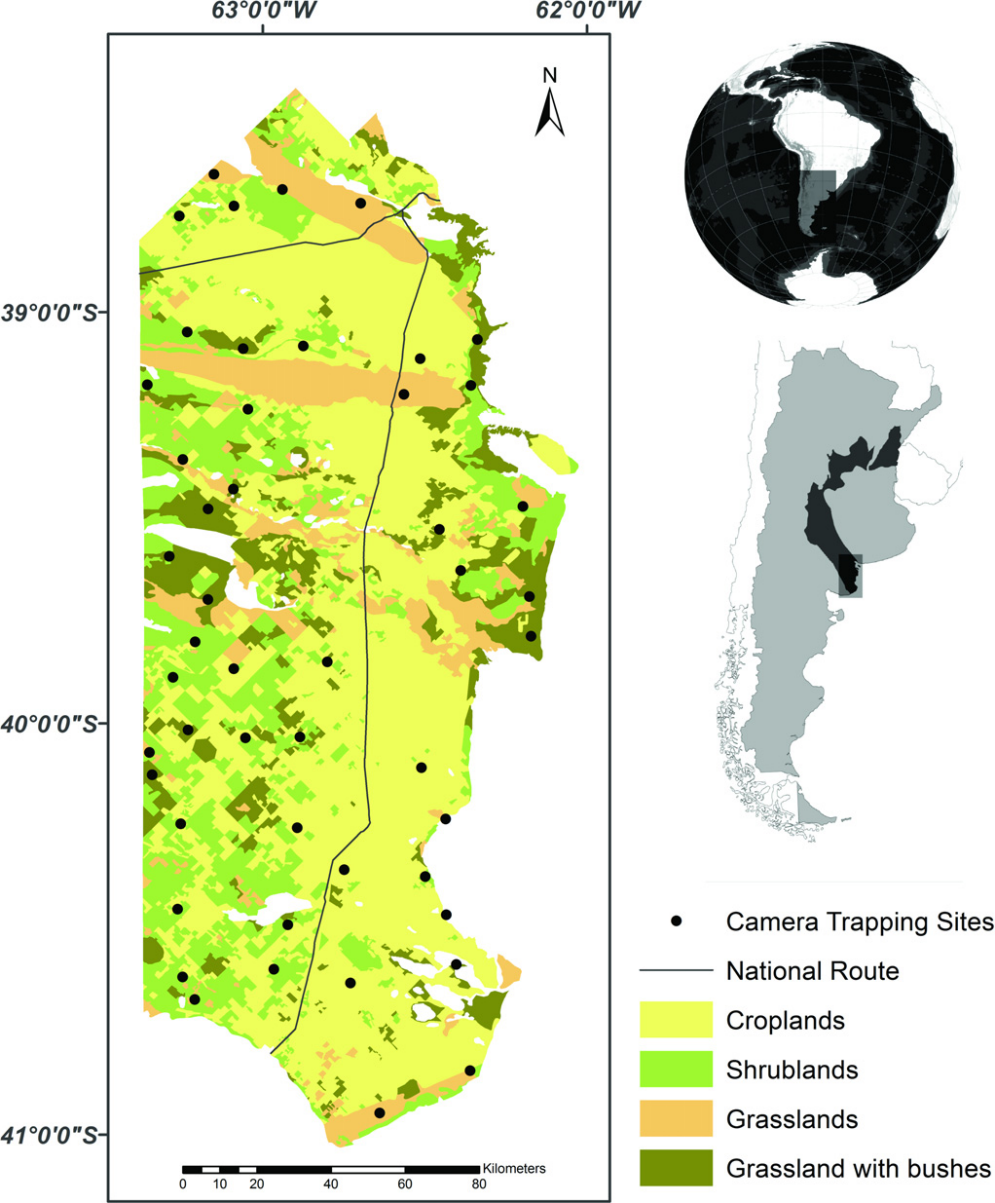
Distribution of camera trapping sites and vegetation categories occurring in the study area. The dark grey area in the map of Argentina represents the Espinal ecoregion.

Fieldwork was conducted in an area of 27,300 km^2^ located in central Argentina and corresponding to the southernmost counties of Buenos Aires province (Fig 1). The study area belongs to the Argentine Espinal ecoregion and is characterized by a template, semiarid climate, where aridity increases towards the west and south [39–41]. The mean annual temperature is 15.3°C. The annual precipitation varies from 350 to 550 mm and concentrates in spring and autumn. The topography is mostly flat and the natural vegetation is characterized by xerophytic deciduous woodlands, prairies dominated by grasslands, and prairies intermixed with extensive shrublands (henceforward, grassland with scrubs). This region has experienced a marked transformation during the last decades due to the increase of agriculture and ranching activities, which are the most important regional sources of income [40]. This habitat alteration and fragmentation processes have converted the original landscape into a mosaic of croplands and pastures with residual patches of original vegetation.

### Data collection

We conducted three camera trapping surveys from January to March in 2011, 2012, and 2013. To randomize the spatial arrangement of the sampling station, we used a GIS layer of the entire study area to draw 100 random points with a distance among them of at least 6 km. We chose this threshold based on the home range size of puma, the largest carnivore of the community. Then, we deployed cameras in 49 of those points (hereafter “sites”) because of logistical restrictions and adjustment of the survey design to the number of cameras available. Each site was composed by an arrangement of 5 cameras stations forming a square with one camera in each vertex (spaced approximately 1.5 km) and one in the center. Each survey was conducted for 35 days (range 25–45) and all cameras were operational 24 hours per day. We checked the cameras every 5 days to replace the batteries, film or memory cards and to ensure their proper functioning. If a camera failed, we replaced it. Sampling effort was calculated as the product of the total number of stations by the number of effective days of sampling (omitting those days in which the cameras did not work due to malfunction or damage caused by cattle, climate or other causes) [42] and totaled 7,054 camera trap days.

### Predictive variables

The study area was characterized using 12 variables related with anthropogenic disturbance and landscape composition and degree of fragmentation (Table 1). For this purpose, we used a vector map provided by the National Institute of Agriculture Technology (INTA), and calculated the value of each variable within a buffer area constructed around each camera site. To account for the fact that the perception that carnivores have of the landscape is related to the sizes of their home ranges [43, 44], we used three different buffer radii (1.5 km for skunks; 3 km for Pampas foxes and Geoffroy’s cats; 6 km for puma). The variables representing distances (“distance to main road” and “distance to urban settlements”) were calculated independently from the buffer size as the Euclidean distance from the camera sites to the landscape element of interest. “Ranch density” was calculated as the number of rural properties intersected by the corresponding buffer divided by buffer area, using a cadastral map of the study area provided by INTA. To describe the level of alteration of the most preserved portions of the region we pooled all natural vegetation categories (except “cropland”) in a new category (“natural vegetation”) and calculated several fragmentation and representativeness indices (Table 1) using the software Fragstat 4.1®.

**Table 1:** Description of the variables used to evaluate the response of four carnivore species to the landscape composition and configuration in the Espinal of the Buenos Aires province, Argentina.

Type of variable	Variable ID (unit)	Description	Transformation
Level of anthropization	DC (n° of farms/km^2^)	Farm density. Number of properties per km^2^	Logarithmic
	DR (km)	Distance in km from the site to the route	Logarithmic
	DL (km)	Distance in km from the site to the closest urban settlement	Logarithmic
Landscape composition	PC	Proportion of the buffer area occupied by the category “cropland”	Angular
	PM	Proportion of the buffer area occupied by the category “shrubland”	Angular
	PP	Proportion of the buffer area occupied by the category “grassland”	Angular
	PPA	Proportion of the buffer area occupied by the category “grassland with bushes”	Angular
Landscape fragmentation	DBC (km/km^2^)	Cropland edge density calculated as the total length of the edges between “croplands” and the other categories divided by the buffer area.	Logarithmic
	TPC (km^2^)	Average area of the patches corresponding to “croplands”	Logarithmic
	DPN (n° parches/km^2^)	Density of the parches corresponding to the category “natural”^a^	Logarithmic
	BTN (km) LSIN (km/km^2^)	Total length of the edges corresponding to the category “natural” landscape shape index	Logarithmic Logarithmic

^a^ The category “natural” was obtained by pooling all vegetation categories, except “cropland”.

### Statistical analysis

To identify the factors affecting the intensity of use of habitat by carnivores, we used the number of independent photographic events obtained in each site as a dependent variable. We considered as independent events all those photos with a temporal separation of at least 30 minutes from each other. Prior to the analysis, all variables were normalized using the angular transformation ($arcsine\sqrt{p}$) for the proportional variables and a logarithmic transformation for all the others (Table 1) [45]. We applied a Principal Component Analysis (PCA) with the objective of reducing the number of variables and the possible effect of multicollinearity among them as well as to obtain linear combinations of these variables describing the prevailing landscape gradients of the study area [46]. Then, we applied a varimax normalized rotation to the set of principal components with eigenvalues > 1, to produce simpler and more interpretable ecological gradients [46].

We used the Akaike Information Criterion adjusted for small samples (AICc) [47] as a measure of relative level of empirical support by each candidate model, with the top-ranking model being the one with the highest Akaike weight (w_i_). Model selection started evaluating two alternative types of potential responses of carnivores to each landscape gradient independently and comparing them to the null model, i.e. the model fitted using the number of sampling days per site as the only variable. To avoid applying overly complex models, we only evaluated linear (y = β_0_+ β_1_x) and quadratic (y = β_0_ + β_1_x + β_2_x^2^) responses to each gradient extracted from the PCA. In all cases we used negative-binomial generalized linear models [48]. To take into account differences in the sampling effort across the sites, we incorporated in all models the natural logarithm of the sampling days as offset. For each species and gradient, we identified the best fitted curve and carried it forward to subsequent analyses, using Akaike weights (w_i_) as the model selection criterion [47]. Then we developed multivariate models describing the relationships between each response variable and the ecological gradients extracted from the PCA. Additionally, we used Multimodel Inferences (MI) to assess the magnitude of the effects of predictors on the response variables. This procedure estimates a weighted average across all models based on their respective weights [47]. All the statistical analyses were performed in R [49]. For the PCA we used the “principal” function (package “psych”), negative-binomial generalized linear models were fitted using the “glm.nb” function (package “MASS”), and the function “dredge” (package “MuMIn”) was used for model selection and MI.

## Results

We obtained a total of 858 events of the four carnivore species (74 for skunks, 546 for Pampas foxes, 193 for Geoffroy’s cats, and 45 for pumas), with an average of 18 carnivore events per site (range = 0–101 events).

### Landscape gradients

**Fig 2:**
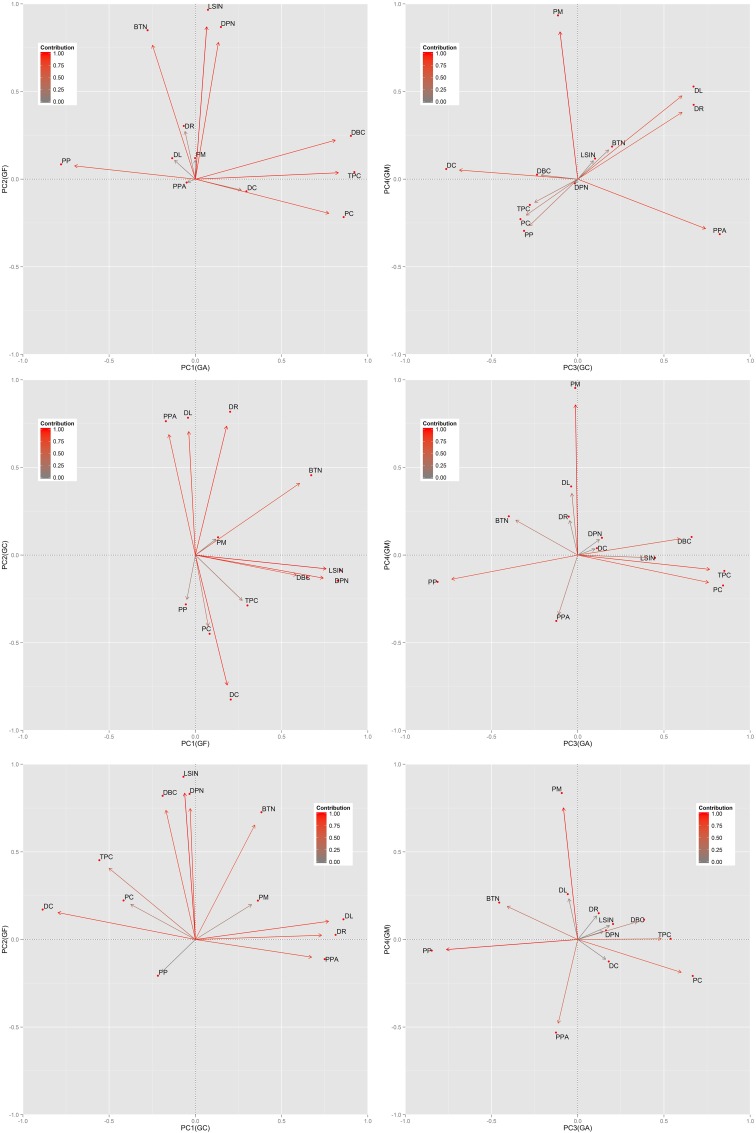
Biplots showing the contribution of each variable to each component extracted throughout Principal Component Analysis (CPA) with normalized Kaiser-varimax rotation for each of the buffer sizes constructed around the sites in the Espinal of Argentina where camera trap data were collected. The upper pair of biplots corresponds to the buffer of 1.5 km, the middle pair corresponds to the buffer of 6 km and the pair below corresponds to the buffer of 6 km. See Table 1 for variable description.

In all cases, the four components (environmental gradients) extracted by the PCA collectively explained more than 80% of the total variance (Table 2). Regardless the size of the buffer used, the principal components extracted were composed by almost the same group of variables, and they showed differences only for two variables related to landscape fragmentation (DBC and TPC; Table 2). One of the gradients, hereafter “Anthropization Gradient or GA”, was essentially related to areas with a high proportion of croplands in the landscape, demonstrated by the association of PC, TPC, and DBC (Table 2 and Fig 2). Also, this component was negatively related with PP. A second component, “Conservation Gradient or GC”, was positively associated with DR, DL, and PPA while negatively related with DC (Table 2 and Fig 2), thus pooling those sites far from the main route and from the urban settlements and with larger ranches. The third component extracted, “Fragmentation Gradient” or GF, was entirely arranged by the fragmentation indices calculated over the “natural” vegetation category (DPN, BTN, and LSIN) (Table 2 and Fig 2) and defined the sites with a greater fragmentation of pristine habitats. Finally, the “Shrubland Gradient or GM” pooled the sites with a high proportion of shrubland vegetation (PM) (Table 2 and Fig 2).

**Table 2:** Cross-loading values of the variables for the four principal components (PC) extracted throughout a Principal Component Analysis (CPA) with normalized Kaiser-varimax rotation for each of the buffer sizes constructed around the sites in the Espinal of Argentina where camera trap data were collected.

Variable	Buffer size											
	1,5 km				3 km				6 km			
	PC1 (GA)	PC2 (GF)	PC3 (GC)	PC4 (GM)	PC1 (GF)	PC2 (GC)	PC3 (GA)	PC4 (GM)	PC1 (GC)	PC2 (GF)	PC3 (GA)	PC4 (GM)
**DC**	0.30	-0.07	**-0.76**	0.06	0.21	**-0.82**	0.11	0.04	**-0.89**	0.17	0.18	-0.13
**DR**	-0.07	0.30	**0.67**	0.42	0.20	**0.82**	-0.05	0.22	**0.81**	0.03	0.12	0.15
**DL**	-0.13	0.12	**0.67**	0.53	-0.04	**0.78**	-0.04	0.39	**0.86**	0.12	-0.06	0.26
**PC**	**0.86**	-0.22	-0.33	-0.23	0.08	-0.45	**0.84**	-0.17	-0.42	0.22	**0.67**	-0.21
**PM**	0.00	0.12	-0.11	**0.93**	0.13	0.10	-0.01	**0.95**	0.36	0.22	-0.09	**0.84**
**PP**	**-0.78**	0.08	-0.31	-0.30	-0.06	-0.28	**-0.81**	-0.15	-0.22	-0.21	**-0.85**	-0.06
**PPA**	-0.05	-0.02	**0.82**	-0.31	-0.17	**0.76**	-0.12	-0.38	**0.75**	-0.11	-0.13	-0.53
**DBC**	**0.90**	0.25	-0.24	0.03	0.65	-0.13	**0.66**	0.10	-0.19	**0.82**	0.38	0.11
**TPC**	**0.92**	0.04	-0.28	-0.15	0.30	-0.29	**0.85**	-0.09	**-0.56**	0.45	0.54	0.00
**DPN**	0.15	**0.87**	-0.02	-0.02	**0.82**	-0.15	0.14	0.10	-0.03	**0.83**	0.16	0.05
**BTN**	-0.28	**0.85**	0.20	0.19	**0.67**	0.46	-0.40	0.22	0.38	**0.73**	-0.46	0.21
**LSIN**	0.07	**0.97**	0.10	0.12	**0.84**	-0.09	0.45	-0.02	-0.07	**0.93**	0.20	0.09
**%V**	**27**	**49**	**70**	**84**	**21**	**47**	**72**	**84**	**30**	**56**	**73**	**83**

Bold values represent the highest absolute loadings for each variable. %V: cumulative percentage variance for each component. GA: Anthropization Gradient, GF: Fragmentation Gradient, GC: Conservation Gradient, GM: Shrubland Gradient. See Table 1 for variable description.

### Carnivore responses

**Fig 3:**
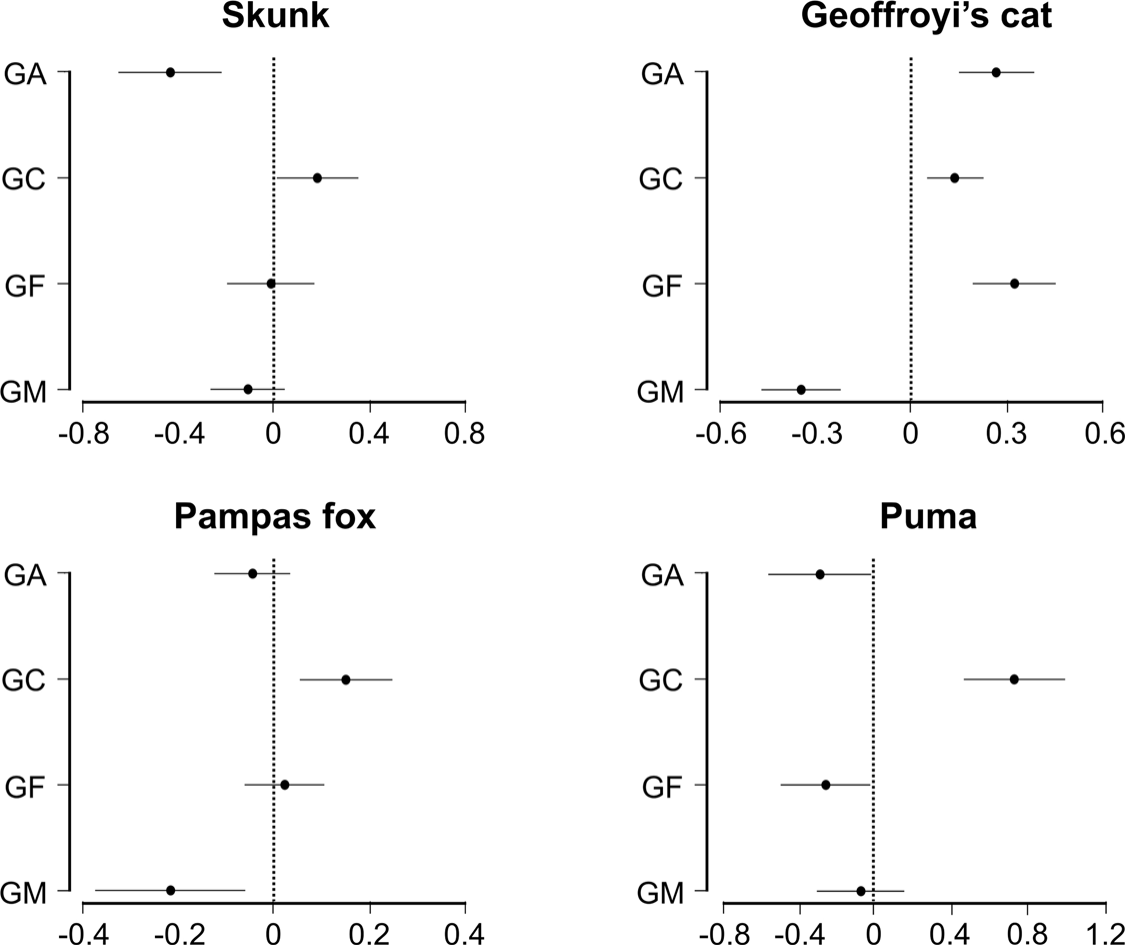
Results of the multimodel inferences for the relationship between carnivores in the Espinal of Argentina and the gradients reflecting four components extracted from a principal component analysis (see Table 2). For each carnivore species the estimated values of the parameters of the average model and its 95% confidence interval are shown. GA: “Anthropization Gradient”, GC: “Conservation Gradient”, GF: “Fragmentation Gradient”, GM: “Shrubland Gradient”.

Positive linear and negative quadratic responses (indicative of a greater use of those sites with intermediate values of the correspondent gradient) were most common among those with the highest probability of selection and significant coefficients. In two cases, a higher support for the positive quadratic response was found, showing a greater use of those sites with extreme values of the correspondent gradient (Figs 2 and 3). The selection process included 6 plausible models in the 95% confidence interval for skunks, Pampas foxes, and pumas, whereas only one model was selected for Geoffroy’s cats (Table 3).

**Table 3:** Summary results of information-theoretic model selection and multimodel inference for the relationships between carnivores in the Espinal of Argentina and four gradients reflecting four components extracted from a principal component analysis of landscape variables.

Species	N	w_i_	Selection probability			
			GA	GC	GF	GM
**Skunk**	6	0.267	0.99	0.64	0.23	0.46
**Geoffroy’s cat**	1	0.931	0.99	0.93	0.99	0.99
**Pampas fox**	6	0.409	0.39	0.95	0.24	0.94
**Puma**	6	0.324	0.69	0.99	0.69	0.27

For each species the table provides the number of models included in the 95% confidence set of models (N), the Akaike weight of the best fitting model (w_i_), and the selection probability for each variable (gradient) used. Selection probabilities of those gradients included in the best fitting model are underlined. GA: “Anthropization Gradient”, GC: “Conservation Gradient”, GF: “Fragmentation Gradient”, GM: “Shrubland Gradient”.

**Fig 4:**
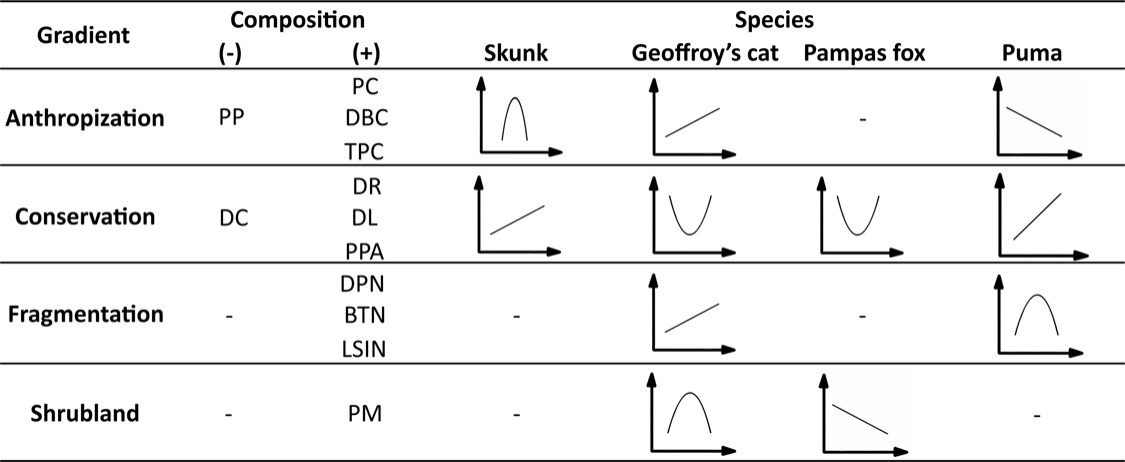
Summary of the significant responses by four carnivore species in the Espinal of Argentina to the four gradients reflecting four components extracted from a principal component analysis. For each gradient we report its association (positive or negative) to of the corresponding environmental variables and the type of response shown by each carnivore species (i.e., linear or quadratic; positive or negative).

The Conservation Gradient was the only factor forming part of the best fitting models for all the carnivores studied (Table 3; Fig 4) and, consistently, was the only gradient with a significant effect on the intensity of habitat use for the four species (Fig 3). The Conservation Gradient had both the greatest selection probability (Table 3) and the highest coefficient for pumas (Figs 2 and 3). For both pumas and skunks, the effect of this gradient was linear positive, whereas the responses of Geoffroy’s cats and Pampas foxes were positive quadratic (Fig 4).

The Pampas fox was the only carnivore for which the Anthropization Gradient showed neither a high probability of selection (Table 3) nor a significant coefficient in the final model (Fig 3). Puma*s* responded negatively to this gradient while for Geoffroy’s cats the coefficient was positive (Fig 3). The Anthropization Gradient was also the gradient with the highest selection probability for skunks (Table 3), with a comparatively strong negative quadratic effect (Fig 4).

Both the Fragmentation and the Shrubland Gradients showed high selection probabilities and significant coefficients only for two species (Table 3 and Fig 3). The Fragmentation Gradient had a significant, positive effect on the habitat use of Geoffroy’s cats, whereas pumas responded in negative quadratic form (Fig 4). Finally, the Shrubland Gradient had significant, negative coefficients for both Geoffroy’s cats and Pampas foxes (Fig 3). However, the response by Pampas foxes was linear and that by Geoffroy’s cat negative quadratic (Fig 4).

## Discussion

Our study demonstrates that human activities impact all species of carnivores in the Espinal of central Argentina and that this effect varies among species differing in their ecological and behavioral attributes as observed in other agricultural landscapes [14]. Because of these species-specific responses of carnivores, we can expect that human-induced changes will modify the structure of the carnivore community, favoring the most adaptable species.

The fact that we found that the Conservation Gradient was the only factor significantly affecting the use of habitat for all the studied carnivore species shows that in general, the most preserved habitats were the most intensively used by carnivores. However, the responses by the Pampas fox and Geoffroy’s cat indicate that, under certain circumstances, these species can also select highly modified habitats. Although the Anthropization Gradient was the second most influential factor in the Espinal of central Argentina, affecting skunks, Geoffroy’s cats, and pumas, its effect varied across the carnivore community.

The Geoffroy’s cat, the most selective of the four carnivores included in this study, was the only species that was significantly affected by all the environmental gradients we identified. This agrees with our prediction that this felid would show a stricter selection of habitat than a more adaptable carnivore such as the Pampas fox. On the other hand, the combination of responses to different environmental components that we found for the Geoffroy’s cats make it difficult to draw clear conclusions with respect to its habitat associations in the Espinal landscape. The Geoffroy’s cat was the only species showing a preference for areas with intermediate proportions of shrublands, in agreement with observation that the habitats with a certain level of vegetation cover are important for this species [50]. In contrast with our prediction, we also found for the Geoffroy’s cat a direct response to the gradients GA and GF, suggesting that, provided that some shrublands are available, the intensity in the use of a particular site by this species would grow with the increase in its level of disturbance and fragmentation. This result differs from some previous studies where Geoffroy’s cats showed a tendency to select areas with low human presence [27, 51, 52]. However, it has been observed that carnivores living in fragmented landscapes show the capacity to explore different areas in search for food and are not limited to use a particular habitat [53]. This might be the case of the Geoffroy’s cat, in accordance with some authors who found a degree of ecological plasticity that would allow this felid to tolerate human disturbance [54] and even survive in strictly agricultural areas [55]. We speculate that the habitat preferences we observed are the result of this ecological adaptability but also of two additional factors: prey availability and differential hunting pressure. Although very little information exits on the abundance of prey species in the Argentine Espinal, it is possible that a greater availability of small rodents, birds, and hares, *Lepus europaeus* (all important items in the diet of this felid [50]), was attracting the Geoffroy’s cat to disturbed areas. Because intraguild interactions may strong affect carnivore occurrence [4, 56, 57], the fact that Geoffroy’s cats in this region suffer a comparatively lower hunting pressure than Pampas foxes and pumas [58] may also drive this cat to exploit highly disturbed areas in order to avoid intraspecific competition. This hypothesis is indirectly supported by the evidence that pumas can predate on Geoffroy’s cats [59].

Skunks preferred areas with intermediate proportions of open habitats (grasslands and croplands), intermediate levels of fragmentation of croplands, a greater proportion of grassland with bushes, larger ranches, and far from the urban settlements and from the main route. These results are in agreement with our prediction that skunks would select cropland areas located in proximity of more densely vegetated patches. The avoidance we found of the most modified portions of the landscape is also in accordance with the hypothesis by Lantschner et al. [51] that at landscape scale, the habitat utilization of this mephitid would be primarily constrained by a high cover of native vegetation as well as with the conclusion of Castillo and Lucherini [60] that, although these skunks are capable of a certain degree of adaption to anthropogenic modifications, their presence is constrained by the availability of remnants of native vegetation.

As we expected for this species, the effect of the environmental factors on Pampas foxes was the weakest. This conclusion is supported by the facts that only two gradients were significantly associated to the intensity of use of habitat of this canid and that both had a small coefficient. Additionally, its response to the Conservation Gradient is indicative that the species tends to use both the sites with larger farms far from the main route and urban settlements and also sites with opposite characteristics, as long as they have a low proportion of shrublands. This strong association of Pampas foxes to open habitats, such as grasslands has been reported previously [21, 61, 62] and suggests that this preference can be the main factor constraining its distribution at landscape scale even in presence of the levels of hunting pressure such as those that this species suffers in the southernmost part of the Espinal [58].

The results for pumas lent clear support to the prediction that this large predator would avoid the sites with high human presence and prefer the most preserved habitats. However, the response to the Fragmentation Gradient showed by pumas suggests that this species could tolerate some degree of fragmentation of the natural habitats. This contrasts with our expectation but is coherent with the findings by other studies that described this felid as a species with the capacity of using a great diversity of habitat types, including those that are highly disturbed [43, 63]. On the other hand, pumas were positively associated to patches of grassland with shrubs located away from roads and urban settlements (i.e. those described by the Conservation Gradient). This result is consistent with those obtained by a habitat suitability model for the puma in the same landscape and the hypothesis derived by this model that grassland with shrubs is a fundamental habitat for the conservation of the populations of the top predator of the Espinal [33].

Although the effect of habitat loss and fragmentation is a central theme in conservation biology [64, 65], the body of empirical evidence mostly comes from single-species studies. In general, it has been found that the diversity and distribution of carnivores in fragmented agricultural ecosystems are strongly influenced by both local and landscape-scale habitat characteristics [14, 66]. Our results for the fragmented landscapes of central Argentina suggest that fragmentation has a comparatively weaker effect on carnivore occurrence than other factors associated with anthropogenic disturbance, such as loss of natural habitats and their replacement with croplands and human presence. This can be related to the comparatively large mobility and ecological adaptability of carnivores and to the strong hunting pressure they typically suffer, at least in regions where livestock is a main source of income [67, 68]. The wide differentiation we found in interspecific responses to environmental factors is also likely to be indicative of a process of niche segregation that may reflect the need for avoiding intraguild interactions that can have strong effects on carnivore occurrence and abundance [69].

## Conclusion

Understand the complexity involved in the relations between wildlife and the environment is crucial for effective conservation plans. This study shows the importance of multi-species landscape-scale studies to identify habitat components that are necessary for the conservation of healthy mammalian communities. Our multivariate gradient approach revealed that despite the fact that the studied species showed a great heterogeneity in their responses to the landscape gradients, our results are in general agreement with the hypothesis that carnivores tend to concentrate their activities in the most preserved portions of intensively modified landscapes [1, 4]. Specifically, it seems clear that in the regions of central Argentina dominated by cattle ranching, carnivore persistence would be conditioned by the maintenance of certain habitat conditions. This is particularly true for the puma, which used almost exclusively the natural habitats away from areas intensively utilized by humans, which confirms previous results showing that most of this territory is now unavailable for this big cat [33], the top carnivore of most of South America. However, also species considered more adaptable, such as the Pampas fox and the Molina’s hog-nosed skunk have shown some type of negative association to areas with human presence. Even in the case of the Geoffroy’s cat, which has shown a surprising degree of association to open, human-dominated sites, the presence of the shelter offered by natural shrubland patches appears to be essential. It remains unclear the role played in this scenario by direct hunting of carnivores and whether a reduction in this pressure would enable pumas and Pampas foxes to use also more intensely modified habitats. Thus, this should be the object of further studies. Nevertheless, we suggest that the conservation of the native community of carnivores of the Argentine Espinal would benefit from actions encouraging the maintenance of the remaining portions of natural habitats, especially shrublands. Although little is known about this topic, given the fragility of soils of this semiarid ecoregion [40], it is likely that shrublands provide key ecosystem services that may benefit also productive activities.

## Supporting Information

S1 Table List of models fitted for each of the four carnivore species studied. GA: “Anthropization Gradient”, GC: “Conservation Gradient”, GF: “Fragmentation Gradient”, GM: “Shrubland Gradient”, df: degree of freedom, logL: log Likelihood, AICc: Akaike Information Criterion corrected for small sample size, ΔAICc: difference in AICc with the top ranking model for a given species, w_i_: Akaike weight.
(DOCX)
