# Supplementary material for: Species-Specific Responses of Carnivores to Human-Induced Landscape Changes in Central Argentina
Source: PLoS One. 2016 Mar 7;11(3):e0150488. doi: 10.1371/journal.pone.0150488 (PMC4780817; doi:10.1371/journal.pone.0150488)
Supplement: S1 Table — GA: “Anthropization Gradient”, GC: “Conservation Gradient”, GF: “Fragmentation Gradient”, GM: “Shrubland Gradient”, df: degree of freedom, logL: log Likelihood, AICc: Akaike Information Criterion corrected for small sample size, ΔAICc: difference in AICc with the top ranking model for a given species, wi: Akaike weight. (DOCX) [file pone.0150488.s001.docx]

|  | **ID** | **Intercept** | **GA^2^** | **GF** | **GC** | **GM^2^** | **df** | **logL** | **AICc** | **ΔAICc** | **w_i_** |
| --- | --- | --- | --- | --- | --- | --- | --- | --- | --- | --- | --- |
| **Skunk** | 6 | 0.52 | -0.41 | - | 0.18 | - | 4 | -356.20 | 721.33 | 0.00 | 0.27 |
|  | 14 | 0.62 | -0.42 | - | 0.18 | -0.11 | 5 | -355.08 | 721.59 | 0.26 | 0.23 |
|  | 2 | 0.60 | -0.48 | - | - | - | 3 | -358.01 | 722.56 | 1.23 | 0.14 |
|  | 10 | 0.70 | -0.49 | - | - | -0.11 | 4 | -356.97 | 722.87 | 1.54 | 0.12 |
|  | 8 | 0.52 | -0.42 | -0.01 | 0.18 | - | 5 | -356.20 | 723.82 | 2.49 | 0.08 |
|  | 16 | 0.63 | -0.43 | -0.02 | 0.18 | -0.12 | 6 | -355.06 | 724.17 | 2.84 | 0.06 |
|  | 4 | 0.61 | -0.50 | -0.04 | - | - | 4 | -357.91 | 724.75 | 3.41 | 0.05 |
|  | 12 | 0.72 | -0.51 | -0.05 | - | -0.12 | 5 | -356.81 | 725.04 | 3.71 | 0.04 |
|  | 5 | 0.21 | - | - | 0.33 | - | 3 | -365.32 | 737.19 | 15.86 | 0.00 |
|  | 13 | 0.31 | - | - | 0.33 | -0.12 | 4 | -364.17 | 737.28 | 15.95 | 0.00 |
|  | 7 | 0.20 | - | 0.12 | 0.34 | - | 4 | -364.36 | 737.65 | 16.32 | 0.00 |
|  | 15 | 0.30 | - | 0.12 | 0.33 | -0.11 | 5 | -363.32 | 738.07 | 16.74 | 0.00 |
|  | 1 | 0.26 | - | - | - | - | 2 | -372.08 | 748.43 | 27.10 | 0.00 |
|  | 9 | 0.37 | - | - | - | -0.12 | 3 | -370.95 | 748.45 | 27.12 | 0.00 |
|  | 3 | 0.25 | - | 0.10 | - | - | 3 | -371.45 | 749.45 | 28.12 | 0.00 |
|  | 11 | 0.36 | - | 0.10 | - | -0.12 | 4 | -370.40 | 749.73 | 28.40 | 0.00 |
|  |  |  |  |  |  |  |  |  |  |  |  |
|  | **ID** | **Intercept** | **GA** | **GF** | **GC**^2^ | **GM**^2^ | **df** | **logL** | **AICc** | **ΔAICc** | **w_i_** |
| **Geoffroy’s cat** | 16 | 1.48 | 0.26 | 0.31 | 0.13 | -0.36 | 6 | -573.69 | 1161.39 | 0.00 | 0.93 |
|  | 14 | 1.57 | 0.24 | 0.23 | - | -0.31 | 5 | -577.65 | 1166.70 | 5.31 | 0.07 |
|  | 12 | 1.54 | - | 0.28 | 0.11 | -0.39 | 5 | -580.69 | 1172.77 | 11.39 | 0.00 |
|  | 10 | 1.62 | - | 0.21 | - | -0.34 | 4 | -583.71 | 1176.34 | 14.95 | 0.00 |
|  | 13 | 1.63 | 0.23 | - | - | -0.34 | 4 | -585.54 | 1179.99 | 18.60 | 0.00 |
|  | 15 | 1.62 | 0.23 | - | 0.03 | -0.35 | 5 | -585.33 | 1182.06 | 20.67 | 0.00 |
|  | 6 | 1.31 | 0.29 | 0.26 | - | - | 4 | -588.27 | 1185.45 | 24.07 | 0.00 |
|  | 8 | 1.24 | 0.30 | 0.30 | 0.06 | - | 5 | -587.30 | 1185.99 | 24.60 | 0.00 |
|  | 9 | 1.68 | - | - | - | -0.37 | 3 | -590.33 | 1187.19 | 25.80 | 0.00 |
|  | 11 | 1.67 | - | - | 0.02 | -0.38 | 4 | -590.23 | 1189.37 | 27.98 | 0.00 |
|  | 2 | 1.35 | - | 0.23 | - | - | 3 | -596.44 | 1199.41 | 38.02 | 0.00 |
|  | 4 | 1.30 | - | 0.26 | 0.04 | - | 4 | -596.06 | 1201.04 | 39.65 | 0.00 |
|  | 5 | 1.35 | 0.28 | - | - | - | 3 | -597.69 | 1201.91 | 40.52 | 0.00 |
|  | 7 | 1.38 | 0.27 | - | -0.03 | - | 4 | -597.40 | 1203.70 | 42.31 | 0.00 |
|  | 1 | 1.38 | - | - | - | - | 2 | -604.34 | 1212.94 | 51.55 | 0.00 |
|  | 3 | 1.43 | - | - | -0.05 | - | 3 | -603.70 | 1213.93 | 52.55 | 0.00 |
|  |  |  |  |  |  |  |  |  |  |  |  |
|  | **ID** | **Intercept** | **GA^2^** | **GF^2^** | **GC^2^** | **GM** | **df** | **logL** | **AICc** | **ΔAICc** | **w_i_** |
| **Pampas fox** | 11 | 1.90 | - | - | 0.14 | -0.22 | 4 | -721.77 | 1452.49 | 0.00 | 0.41 |
|  | 15 | 1.92 | -0.05 | - | 0.16 | -0.22 | 5 | -720.93 | 1453.32 | 0.83 | 0.27 |
|  | 12 | 1.88 | - | 0.02 | 0.14 | -0.21 | 5 | -721.69 | 1454.84 | 2.35 | 0.13 |
|  | 16 | 1.89 | -0.05 | 0.02 | 0.16 | -0.22 | 6 | -720.80 | 1455.70 | 3.21 | 0.08 |
|  | 3 | 1.85 | - | - | 0.21 | - | 3 | -725.63 | 1457.82 | 5.33 | 0.03 |
|  | 9 | 2.05 | - | - | - | -0.34 | 3 | -725.66 | 1457.87 | 5.38 | 0.03 |
|  | 7 | 1.87 | -0.05 | - | 0.23 | - | 4 | -724.86 | 1458.67 | 6.18 | 0.02 |
|  | 4 | 1.82 | - | 0.03 | 0.21 | - | 4 | -725.41 | 1459.76 | 7.28 | 0.01 |
|  | 10 | 2.04 | - | 0.01 | - | -0.34 | 4 | -725.61 | 1460.18 | 7.69 | 0.01 |
|  | 13 | 2.06 | 0.00 | - | - | -0.35 | 4 | -725.65 | 1460.26 | 7.77 | 0.01 |
|  | 8 | 1.83 | -0.05 | 0.03 | 0.23 | - | 5 | -724.59 | 1460.63 | 8.15 | 0.01 |
|  | 14 | 2.04 | 0.00 | 0.01 | - | -0.34 | 5 | -725.61 | 1462.68 | 10.19 | 0.00 |
|  | 1 | 2.12 | - | - | - | - | 2 | -738.52 | 1481.32 | 28.83 | 0.00 |
|  | 5 | 2.08 | 0.04 | - | - | - | 3 | -737.94 | 1482.44 | 29.95 | 0.00 |
|  | 2 | 2.09 | - | 0.03 | - | - | 3 | -738.35 | 1483.26 | 30.78 | 0.00 |
|  | 6 | 2.06 | 0.04 | 0.03 | - | - | 4 | -737.80 | 1484.55 | 32.06 | 0.00 |
|  |  |  |  |  |  |  |  |  |  |  |  |
|  | **ID** | **Intercept** | **GA** | **GF**^2^ | **GC** | **GM** | **df** | **logL** | **AICc** | **ΔAICc** | **w_i_** |
| **Puma** | 8 | -0.18 | -0.28 | -0.25 | 0.72 | - | 5 | -286.11 | 583.62 | 0.00 | 0.32 |
|  | 4 | -0.08 | - | -0.31 | 0.76 | - | 4 | -287.88 | 584.67 | 1.05 | 0.19 |
|  | 6 | -0.38 | -0.33 | - | 0.69 | - | 4 | -287.96 | 584.83 | 1.21 | 0.18 |
|  | 16 | -0.18 | -0.29 | -0.24 | 0.70 | -0.08 | 6 | -285.88 | 585.75 | 2.14 | 0.11 |
|  | 14 | -0.38 | -0.34 | - | 0.66 | -0.11 | 5 | -287.50 | 586.39 | 2.77 | 0.08 |
|  | 12 | -0.08 | - | -0.30 | 0.74 | -0.06 | 5 | -287.77 | 586.94 | 3.32 | 0.06 |
|  | 2 | -0.32 | - | - | 0.74 | - | 3 | -290.66 | 587.84 | 4.23 | 0.04 |
|  | 10 | -0.32 | - | - | 0.72 | -0.09 | 4 | -290.37 | 589.64 | 6.02 | 0.02 |
|  | 13 | -0.19 | -0.46 | - | - | -0.24 | 4 | -298.68 | 606.27 | 22.66 | 0.00 |
|  | 15 | -0.10 | -0.43 | -0.10 | - | -0.23 | 5 | -298.45 | 608.29 | 24.68 | 0.00 |
|  | 5 | -0.16 | -0.46 | - | - | - | 3 | -300.90 | 608.33 | 24.72 | 0.00 |
|  | 7 | -0.02 | -0.41 | -0.15 | - | - | 4 | -300.31 | 609.53 | 25.92 | 0.00 |
|  | 11 | 0.10 | - | -0.22 | - | -0.20 | 4 | -302.36 | 613.63 | 30.01 | 0.00 |
|  | 9 | -0.10 | - | - | - | -0.24 | 3 | -303.67 | 613.87 | 30.26 | 0.00 |
|  | 3 | 0.16 | - | -0.27 | - | - | 3 | -303.77 | 614.08 | 30.46 | 0.00 |
|  | 1 | -0.06 | - | - | - | - | 2 | -305.68 | 615.62 | 32.00 | 0.00 |
